# Gut microbiota in patients with obesity and metabolic disorders — a systematic review

**DOI:** 10.1186/s12263-021-00703-6

**Published:** 2022-01-29

**Authors:** Zhilu XU, Wei JIANG, Wenli HUANG, Yu LIN, Francis K.L. CHAN, Siew C. NG

**Affiliations:** 1 Department of Medicine and Therapeutics, Institute of Digestive Disease, State Key Laboratory of Digestive Diseases, LKS Institute of Health Science, The Chinese University of Hong Kong, Hong Kong, China; 2 Center for Gut microbiota research, Faculty of Medicine, The Chinese University of Hong Kong, Hong Kong, China; 3 Microbiota Innovation Centre (MagIC Centre), Hong Kong, China

**Keywords:** Obesity, Metabolic disorder, Microbiota

## Abstract

**Background:**

Previous observational studies have demonstrated inconsistent and inconclusive results of changes in the intestinal microbiota in patients with obesity and metabolic disorders. We performed a systematic review to explore evidence for this association across different geography and populations.

**Methods:**

We performed a systematic search of MEDLINE (OvidSP) and Embase (OvidSP) of articles published from Sept 1, 2010, to July 10, 2021, for case–control studies comparing intestinal microbiome of individuals with obesity and metabolic disorders with the microbiome of non-obese, metabolically healthy individuals (controls). The primary outcome was bacterial taxonomic changes in patients with obesity and metabolic disorders as compared to controls. Taxa were defined as “lean-associated” if they were depleted in patients with obesity and metabolic disorders or negatively associated with abnormal metabolic parameters. Taxa were defined as “obesity-associated” if they were enriched in patients with obesity and metabolic disorders or positively associated with abnormal metabolic parameters.

**Results:**

Among 2390 reports screened, we identified 110 full-text articles and 60 studies were included. Proteobacteria was the most consistently reported obesity-associated phylum. Thirteen, nine, and ten studies, respectively, reported *Faecalibacterium*, *Akkermansia*, and *Alistipes* as lean-associated genera. *Prevotella* and *Ruminococcus* were obesity-associated genera in studies from the West but lean-associated in the East. *Roseburia* and *Bifidobacterium* were lean-associated genera only in the East, whereas *Lactobacillus* was an obesity-associated genus in the West.

**Conclusions:**

We identified specific bacteria associated with obesity and metabolic disorders in western and eastern populations. Mechanistic studies are required to determine whether these microbes are a cause or product of obesity and metabolic disorders.

**Supplementary Information:**

The online version contains supplementary material available at 10.1186/s12263-021-00703-6.

## Introduction

Obesity-related metabolic disorders, including type 2 diabetes (T2DM), cardiovascular diseases, and non-alcoholic fatty liver disease (NAFLD), affect 13% of the population and result in 2.8 million deaths each year [1, 2], and are a significant socioeconomic burden to society. Pathophysiology of obesity and metabolic disorders is multi-factorial, and currently, therapies are limited. The role of intestinal microbiota in patients with obesity and metabolic disorders have been extensively studied in the past decade. Humanized mouse models showed that the microbiome in obese subjects appeared to be more efficient in harvesting energy from the diet and may thereby contribute to the pathogenesis of obesity [3, 4]. However, observational studies reported inconsistent and inconclusive changes of intestinal microbiota in patients with obesity and metabolic disorders [5]. For instance, the Firmicutes and Bacteriodetes ratio (F/B ratio) is not a reproducible marker across human cohorts [6].

Microbial-based therapies such as probiotics aiming to reshape the gut microbial ecosystem have been increasingly explored in the treatment of obesity-related metabolic disorders [7, 8]. Traditional probiotics, primarily consisting of *Lactobacillus* and *Bifidobacterium* have been shown to elicit weight loss in subjects with obesity yet the effect sizes were small with large variations of efficacy among different studies [9]. Emerging evidence showed that *Akkermansia muciniphila* was depleted in patients with obesity-related metabolic disorders. These results have led to mechanistic studies and clinical trials to test its efficacy in the management of obesity and metabolic disorders [10].

Age, geography, and dietary patterns largely affect the gut microbiome [11–13]. The gut microbiota of vegetarians was dominated by *Clostridium* species [14] whereas subjects who mainly consumed fish and meat had high level of *F. prausnitzii* [15]. In recent years, the prevalence of childhood obesity has increased sharply. However, only limited data has issued the function and structure of gut microbiota in children and adolescents with obesity [16].

We have therefore conducted a systematic review of case–control studies evaluating the microbiota in patients with obesity and metabolic disorders compared to lean, healthy controls to summarize the current evidence in the relationship between individual members of the microbiota and obesity. We aimed to identify novel candidates as live biotherapeutics to facilitate the treatment of obesity and metabolic disorders.

## Materials and methods

### Search strategy

This systematic review was performed in accordance with the PRISMA 2009 guidelines [17]. We performed a systematic search of MEDLINE (OvidSP) and Embase (OvidSP) of articles published from Sept 1, 2010 to July 10, 2021 to identify case-control studies comparing gut microbiota in patients with obesity and metabolic disorder and non-obese, metabolically healthy controls. Search strategy is shown in the Appendix.

### Study selection and patient population

Studies were included if they were (1) case–control studies comparing gut microbiota in patients with obesity and metabolic disorders and non-obese, metabolically healthy individuals (controls); (2) intestinal microbiota was assessed by next-generation sequencing (NGS; 16s rRNA amplicon or shotgun metagenomic sequencing); and (3) obesity was defined based on body mass index (BMI) ≥ 30kg/m^2^ and metabolic disorders including type 2 diabetes mellitus, non-alcoholic fatty liver disease, cardiovascular disease, and metabolic syndrome were diagnosed according to respective guidelines (Table 1). Studies from all age groups were included. Studies were excluded if they were (1) case reports, reviews, meta-analyses, re-analysis of public datasets, or conference abstracts, (2) without data for individual bacterial groups, (3) not in English, and (4) not a case–control design. Studies of genetic-associated obesity such as Prader–Willi syndrome were also excluded.

**Table 1:** General Characteristics of included studies

First author, year	Country	Ethnicity	Disease	Sample size (case)	Sample size (control)	Age (years)	Sample	Sequencing Method	Definition of obesity	Definition of metabolic diseases
Andoh, 2016 [18]	Japan	Asian	OB	10	10	31–58	Stool	16s rRNA (V3–V4)	BMI ≥ 35.7 kg/m^2^	NA
Bai, 2019 [19]	USA	Caucasian	OB	43	224	7–18	Stool	16s rRNA (V4)	BMI > 95th percentile	NA
Chen, 2020 [20]	China	Asian	OB	28	23	6–11	Stool	16s rRNA (V4)	Body mass index cut-offs for overweight and obesity in Chinese children and adolescents aged 2–18 years*	NA
Da Silva, 2020 [21]	Trinidad	Asian/Black	OB	21	30	6–14	Stool	16s rRNA (not specified)	> 97th percentile	NA
Gao, 2018 [22]	China	Asian	OB	167(OB: *n* = 145;OW: *n* = 22)	25	NW:25.4 ± 3.2; OW:30.1 ± 11.2; OB:29.2 ± 11.4	Stool	16s rRNA (V4)	NA	NA
Gao, 2018 [23]	China	Asian	OB	39	38	OB: 6.8 ± 1.6; NW: 6.0 ± 2.7	Stool	16S rRNA (V3–V4)	BMI ≥ 30 kg/m^2^	NA
Haro, 2016 [24]	Spain	Caucasian	OB	49	26	Men: 61.15 ± 1.27; Women: 60.31 ± 1.40	Stool	16s rRNA (V4)	BMI ≥ 30 kg/m^2^	NA
Houttu, 2018 [25]	Finland	Caucasian	OB	47	52	30 ± 5	Stool	16s rRNA (not specified)	BMI ≥ 30 kg/m^2^	NA
Hu, 2015 [26]	Korea	Asian	OB	67	67	13–16	Stool	16s rRNA (V1–V3)	BMI ≥30 kg/m^2^ or ≥ 99th BMI percentile	NA
Kaplan, 2019 [27]	USA	Caucasian	OB	294	293	18–74	Stool	16s rRNA (V4)	BMI ≥ 30 kg/m^2^	NA
Liu, 2017 [28]	China	Asian	OB	72	79	OB:23.6 ± 3.7; NW:23.2 ± 1.8	Stool	Metagenomics/16S rRNA (V3–V4)	BMI ≥ 30 kg/m^2^	NA
Lopez-Contreras, 2018 [29]	Mexico	Hispanic/Latino	OB	71	67	6–12	Stool	16s rRNA (V4)	BMI ≥ 95th percentile	NA
Lv, 2019 [30]	China	Asian	OB	9	19	18–27	Stool	16S rRNA (V3–V4)	OW, BMI ≥ 24 kg/m^2^ OB, BMI ≥ 28 kg/m^2^	NA
Mendez-Salazar, 2018 [31]	Mexico	Hispanic/Latino	OB	12	12	9–11	Stool	16s rRNA (V3–V4)	BMI *z*-score≥ +2 standard deviations	NA
Nardelli, 2020 [32]	Italy	Caucasian	OB	19	16	20–80	Duodenal biopsies	16s rRNA V4–V6	BMI ≥ 30 kg/m^2^	NA
Blasco, 2017 [33]	Spain	Caucasian	OB	14	13	30–65	Stool	Metagenomics	BMI ≥ 30 kg/m^2^	NA
Davis, 2017 [34]	UK	Caucasian	OB	54 (OB/OW:*n* = 27)	27	19–70	Stool	Metagenomics/16s rRNA (V4)	NA	NA
Dominianni, 2015 [35]	USA	Caucasian	OB	11	82	30–83	Stool	16S rRNA (V3–V4)	BMI ≥ 25 kg/m^2^	NA
Escobar, 2015 [36]	Colombia	Hispanic/Latino	OB	NA	30	21–60	Stool	16s rRNA (V1–V3)	BMI ≥ 30.0 kg/m^2^	NA
Kasai, 2015 [37]	Japan	Asian	OB	33	23	Non-obese:45.6 ± 9.6; Obese:54.4 ± 8.2	Stool	16s rRNA (V3–V4)	BMI ≥ 25kg/m^2^	NA
Nirmalkar, 2018 [38]	Mexico	Hispanic/Latino	OB	96	76	6–18	Stool	16s rRNA V3	BMI ≥ 95th percentile	NA
Ottosson, 2018 [39]	Sweden	Caucasian	OB	NA	NA	> 18	Stool	16s rRNA (V1–V3)	BMI > 30.0 kg/m^2^	NA
Peters, 2018 [40]	USA	Caucasian	OB	388	211	18–86	Stool	16s rRNA V4	BMI ≥ 30 kg/m^2^	NA
Ppatil, 2012 [41]	India	Asian	OB	5	5	21–62	Stool	16s rRNA (not specified)	BMI: 25–53 kg/m^2^	NA
Rahat-Rozenbloom,2014 [42]	Canada	Caucasian	OB	11	11	> 17	Stool	16s rRNA (V6)	BMI > 25 kg/m^2^	NA
Riva, 2017 [43]	Italy	Caucasian	OB	42	36	9–16	Stool	16s rRNA V3–V4	BMI *z*-score	NA
Vieira-Silva, 2020 [44]	Belgium	Caucasian	OB	474	414	18–76	Stool	Metagenomics	BMI ≥ 30 kg/m^2^	NA
Ville, 2020 [45]	USA	Hispanic/Latino	OB	6	39	0.5–1	Stool	16s rRNA V4	BMI ≥ 95th percentile	NA
Yasir, 2015 [46]	France/Saudi Arabia	Caucasian/Asian	OB	21	25	≥ 18	Stool	16s rRNA (V3–V4)	BMI ≥ 30.0 kg/m^2^	NA
Yun, 2017 [47]	Korea	Asian	OB	745 (OB:*n* = 419; OW: *n* = 326)	529	> 18	Stool	16s rRNA V3–V4	BMI ≥ 25 kg/m^2^	NA
Zacarias, 2018 [48]	Finland	Caucasian	OB	29 (OB: *n* = 11, OW: *n* = 18)	25	NW:29.6 ± 4.2; OW:30.4 ± 3.6; OB:29.6 ± 2.3	Stool	16s rRNA V3–V4	BMI≥30 kg/m^2^	NA
Allin, 2018 [49]	Denmark	Caucasian	T2DM	134	134	55–68	Stool	16s rRNA (V4)	NA	Fasting plasma glucose of 6.1–7.0 mmol/l or HbA1c of 42–48 mmol/mol [6.0–6.5%]
Barengolts, 2018 [50]	USA	Black	T2DM	73	20	35–70	Stool	16s rRNA (V3–V4)	NA	HbA1c of 6.5–7.4%
Leite, 2017 [51]	Brazil	Hispanic/Latino	T2DM	20	22	36–75	Stool	16s rRNA (V3–V4)	NA	Fasting blood glucose levels ≥ 126 mg/dL
Qin, 2012 [52]	China	Asian	T2DM	170	174	25–86	Stool	Metagenomics	NA	NA
Karlsson, 2013 [53]	Sweden	Caucasian	T2DM	102	43	70	Stool	Metagenomics	NA	Glucose metabolism impairment: fasting hyperglycaemia (fasting venous plasma glucose ≥ 6.1 and < 7.0 mmol/L) or IGT (fasting venous plasma glucose <7 mmol/L, ≥ 7.8 and < 11.1 mg/dL 2 h after OGTT) or new onset T2DM (fasting glucose ≥ 7 mmol/L or ≥ 11.1 mmol/L 2 h after OGTT); Arterial hypertension (AH) (systolic/diastolic blood pressure level of 140/90–159/99 mmHg).
Larsen, 2010 [54]	Denmark	Caucasian	T2DM	18	18	31–73	Stool	16s rRNA (V4–V6)	NA	The diabetic group had elevated concentration of plasma glucose as determined by OGTT. Non-diabetic group based on the measurements of baseline glucose and biochemical analysis of blood samples.
Ahmad, 2019 [55]	Pakistan	Asian	T2DM	40	20	25–55	Stool	16s rRNA (V3–V4)	NA	NA
Koo, 2019 [56]	China, Malaysia, and India	Asian	T2DM	22	13	22–70	Stool	16s rRNA (V3–V6)	waist circumference ≥ 90 cm in men and ≥ 80 cm in women	DM were excluded by the absence of impaired glucose tolerance on fasting blood glucose.
Sroka-oleksiak, 2020 [57]	Poland	Caucasian	T2DM	OB: *n* = 17;OB+T2DM: *n* = 22)	27	20–70	Duodenal biopsies	16s rRNA (V3–V4)	BMI >35 kg/m^2^	NA
Thingholm, 2019 [58]	Germany	Caucasian	T2DM	OB: *n* = 494;OB+T2DM: *n* = 153)	633	21–78	Stool	Metagenomics/16s rRNA (V1–V2)	BMI > 30.0 kg/m^2^	Fasting glucose level ≥ 125 mg/dl
Zhao, 2019 [59]	China	Asian	NAFLD	OB: *n* = 18;NAFLD: *n* = 25)	15	9–17	Stool	Metagenomics	BMI ≥ 95th percentile	NA
Jiang, 2015 [60]	China	Asian	NAFLD	35	30	22–72	Stool	16s rRNA (V3)	NA	Based on evidence of hepatic steatosis via either imaging or histology
Shen, 2017 [61]	Chinese	Asian	NAFLD	25	22	> 18	Stool	16s rRNA (V3–V5)	NA	NAFLD can be diagnosed by the presence of three findings: (i) the histological findings of liver biopsy are in accord with the pathological diagnostic criteria of fatty liver disease. (ii) there is no history of alcohol drinking habit or the ethanol intake per week was less than 140 g in men (70 g in women) in the past 12 months; (iii) specific diseases that could lead to steatosis, such as viral hepatitis, drug-induced liver disease, total parenteral nutrition, Wilson’s disease, and autoimmune liver disease, can be excluded.
Sobhonslidsuk, 2018 [62]	Thailand	Asian	NASH	16	8	NASH:59.8 ± 9.6; control:43.4 ± 6.8	Stool	16s rRNA (V3–V4)	NA	NAFLD activity score ≥ 5
Wang, 2016 [63]	China	Asian	NAFLD	43	83	33–61	Stool	16s rRNA (V3)	NA	Evidence of fatty liver upon ultrasonography
Li, 2018 [64]	China	Asian	NAFLD	30	37	18–70	Stool	16s rRNA (V4)	NA	The diagnosis of NAFLD was based on the following criteria: (i) abdominal ultrasonography indicated a fatty liver; (ii) the patient’s alcohol consumption was less than 20 g/day and 10 g/day for male for female.
Nistal, 2019 [65]	Spain	Caucasian	NAFLD	53	20	20–60	Stool	16S rRNA (V3–V4)	NA	An NAFLD diagnosis was established by clinical, analytical criteria (liver function test) and from ultrasonographic data when steatosis was detected.
Yun, 2019 [66]	Korea	Asian	NAFLD	76	192	43.6 ± 8.2	Stool	16s rRNA (V3–V4)	BMI ≥ 25 kg/m^2^	U/S findings suggestive of fatty liver disease
Michail, 2015 [67]	USA	Caucasian	NAFLD	24	26	13.2 ± 3.8	Stool	16s rRNA (not specified)	BMI ≥ 95th percentile	Ultrasound findings and elevated transaminases suggestive of NAFLD
Zhu, 2013 [68]	USA	Caucasian	NASH	47	16	< 18	Stool	16s rRNA (not specified)	BMI ≥ 95th percentile	NAFLD activity score≥ 5
Chavez-Carbaja, 2019 [69]	Mexico	Hispanic/Latino	MS	42	25	18–59	Stool	16s rRNA (V4)		At least three of the following issues: waist greater than 102 cm in males or 82 cm in females, triglycerides levels greater or equal to 150 mg/dl, HDL cholesterol levels less than 40 mg/dl in males or less than 50 mg/dl in females, blood pressure greater or equal to 130/85 mmHg and a fasting blood glucose level higher or equal to 100 mg/dl.
De La Cuesta-Zuluaga, 2018 [70]	Colombia	Hispanic/Latino	MS	291	151	18–62	Stool	16s rRNA (V4)	BMI ≥ 30.0 kg/m^2^	At least two of the following conditions: systolic/diastolic blood pressure ⩾130/85 mm Hg or consumption of antihypertensive medication; fasting triglycerides ⩾150 mg/dl; HDL ≤ 40 mg /dl (men), ≤ 50 mg/dl (women) or consumption of lipid-lowering medication; fasting glucose ⩾ 100 mg/dl or consumption of antidiabetic medication; HOMA-IR 43, and hs-CRP 43 mg L^−1^.
Gallardo-Becerra, 2020 [71]	Mexico	Hispanic/Latino	MS	17	10	7–10	Stool	16s rRNA (V4)	BMI> 95^th^ percentile	At least two of the following metabolic traits: (1) triglycerides > 1.1 mmol/L (100 mg/dL); (2) HDL cholesterol < 1.3 mmol/L (50 mg/dL); (3) glucose > 6.1 mmol/L (110 mg/dL); (4) systolic blood pressure > 90th percentile for gender, age, and height.
Gozd-Barszczewska, 2017 [72]	Poland	Caucasian	MS	15	5	45–65	Stool	16s rRNA (V3–V5)	BMI ≥ 30.0 kg/m^2^	Lipid profile was assessed based on ESC/EAS Guidelines
Kashtanova, 2018 [73]	Russia	Caucasian	MS	57	35	25–76	Stool	16s rRNA (V3–V4)	BMI ≥ 30 kg/m^2^ and/or waist circumference ≥ 94 cm for men and ≥ 80 cm for women	Glucose metabolism impairment: fasting hyperglycaemia (fasting venous plasma glucose ≥ 6.1 and < 7.0 mmol/L) or IGT (fasting venous plasma glucose < 7 mmol/L, ≥7.8 and < 11.1 mg/dL 2 h after OGTT) or new onset T2DM (fasting glucose ≥ 7 mmol/L or ≥11.1 mmol/L 2 h after OGTT); Arterial hypertension (AH) (systolic/diastolic blood pressure level of 140/90–159/99 mmHg).
Lippert, 2017 [74]	Austria	Caucasian	MS	12	8	58–71	Stool	16s rRNA (V1–V3)	NA	At least two of the following conditions: systolic/diastolic blood pressure ⩾ 130/85 mm Hg or consumption of antihypertensive medication; fasting triglycerides ⩾ 150 mg/dl; HDL ≤ 40 mg/dl (men),≤ 50 mg/dl (women), or consumption of lipid-lowering medication; fasting glucose ⩾ 100 mg/dl or consumption of antidiabetic medication; HOMA-IR 43, and hs-CRP 43 mg L^−1^.
Feinn, 2020 [75]	Italy	Caucasian	NAFLD	44	29	NAFLD: 13.3 ± 3.2; OB without NAFLD: 12.9 ± 2.8	Stool	16s rRNA (V4)	BMI ≥ 95th percentile	Hepatic fat fraction ≥ 5.5%
Li, 2021 [76]	China	Asian	OB	3	3	OB:34.33 ± 0.47; NW:25.67 ± 1.25	Stool	16s rRNA (V3–V4)	BMI≥ 30.0 kg/m2	NA
Yuan, 2021 [77]	China	Asian	MS	65	21	5–15	Stool	16s rRNA (V3–V4)	NA	The presence of at least one of the following metabolic traits: (1) FPG ≥ 5.6 mmol/L; (2) systolic blood pressure ≥ 90th percentile for gender and age; (3) fasting HDL-C < 1.03 mmol/L; and (4) fasting TG ≥ 1.7 mmol/L.

*OW* overweight, *OB* obesity, *T2DM* diabetes mellitus type 2, *NAFLD* non-alcoholic fatty liver disease, *MS* metabolic syndrome, *NASH* non-alcoholic steatohepatitis, *NA* not appliable, *IGT* impaired glucose tolerance

*Refers to a standard developed by the Department of Growth and Development, Capital Institution of Pediatrics, China, to define children of obesity

### Study outcomes

The primary outcome was the bacterial taxonomic changes in patients with obesity and metabolic disorders compared to non-obese, metabolically healthy controls. Secondary outcomes included the changes in bacteria diversity and F/B ratio, subgroup analysis of microbiota changes in adults and children with obesity and metabolic disorders, and in Eastern and Western populations. Data on microbiota community composition were extracted from each study. Taxa were defined as “lean-associated” if they were depleted in patients with obesity and metabolic disorders or negatively associated with abnormal metabolic parameters such as high body mass index (BMI), elevated fasting plasma glucose and elevated serum cholesterol. Taxa were defined as “obesity-associated” if they were enriched in patients with obesity and metabolic disorders or positively associated with abnormal metabolic parameters. Taxon at each level (phylum, class, order, family, genus) was only counted once for each study (i.e., if a genus was both depleted in obesity and negatively associate with fat mass in the same study, it was only counted once).

### Eligibility assessment and data extraction

Two authors (JW, HW) independently reviewed studies and excluded based on titles, abstracts, or both to lessen the selection bias and then reviewed selected studies with full text for complete analysis. JW extracted data from studies and entered it into a designated spreadsheet. HW checked the accuracy of this process. The data were re-checked when there was a discrepancy. XZ arbitrated if the discrepancy cannot be resolved by consensus and discussion. The data collected included the following: participant characteristics, including age group, country, types of metabolic disorders, number of patients; types of specimens, microbiota assessment method, microbiome diversity, and Firmicutes/Bacteroides ratio.

### Quality assessment

The Newcastle-Ottawa Scale was applied to assess the quality of included studies. The Newcastle-Ottawa Scale consists of 3 domains (maximum 9 stars); selection (is the case definition adequate, representativeness of the cases, selection of controls, definition of controls); comparability (comparability of baseline characteristics); and exposure (ascertainment of exposure, same method of ascertainment for cases and controls, attrition rate).

## Results

### Study characteristics

**Fig. 1:**
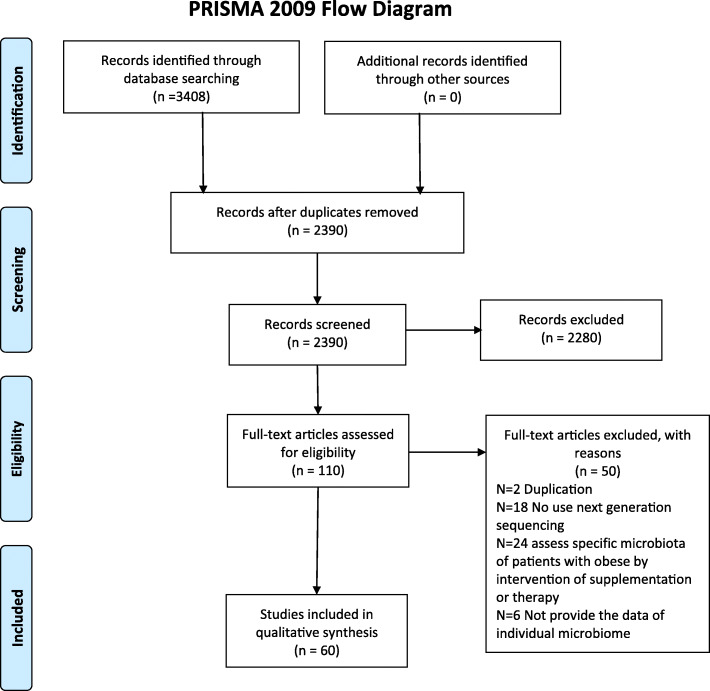
Flowchart of study selection

Overall, 2390 citations were retrieved; 2280 were excluded based on title, abstract, and the availability of full text; 110 articles were subsequently fully reviewed. After further review, 50 full-text articles were rejected (Fig. 1). The final analysis included 60 studies (Table 1). Of these, 44 studies assessed the gut microbiota in adults and 16 in infants, children, and adolescents. Ethnicity of subjects consisted of Asian, Black, Caucasian, Hispanic, or Latino. Fifty-eight out of 60 (96.7%) studies evaluated intestinal microbiota in stool samples and two studies assessed the microbiota in duodenal biopsies. Thirty-two studies involved patients with obesity [18–48, 76], ten involved patients with T2DM [49–58], eleven involved patients with NAFLD or non-alcoholic steatohepatitis (NASH) [59–68, 75], and seven involved patients with metabolic syndrome [69–74, 77]. General characteristics and diagnostic criteria for obesity and metabolic disorders in each study were summarized in Table 1.

### Microbiome assessment methods

Of the 58 studies assessing stool microbiome, 50 studies assessed the gut microbiota by using 16S ribosomal RNA (rRNA) gene sequencing, six used shotgun metagenomic sequencing and two studies applied both 16s rRNA and shotgun metagenomic sequencing. Both studies assessing biopsy microbiome applied 16S rRNA sequencing.

### Primary outcomes

At the phylum level, significant changes of phyla Firmicutes, Bacteroidetes, and Proteobacteria were most reported in obese, metabolic diseased subjects compared with controls. Among 60 studies included, 22 studies reported significant changes in Firmicutes with 15 studies showing phylum Firmicutes were obesity-associated and 7 showing it was lean-associated [18, 21, 23, 28, 29, 32, 34, 42, 43, 45, 46, 48,50, 53–55, 59, 62, 63, 68, 69, 71]; 20 studies reported significant changes in Bacteroidetes with 8 studies showing it was obesity-associated and 12 showing it was lean-associated [20, 23, 29, 31, 32, 35, 37, 43, 46, 55, 57, 59, 61–63, 68, 69, 71, 74, 75]. Fifteen studies reported significant change in Proteobacteria with 13 studies showing it was obesity-associated and 2 showing it was lean-associated [19, 20, 22, 29, 31, 32, 45, 46, 55, 59, 61, 65, 68, 69, 71]. Studies consistently reported that Fusobacteria as obesity-associated taxa (*n* = 5) [18, 20, 22, 32, 61], Actinobacteria was a lean-associated taxa (*n* = 7) [20, 23, 32, 45, 62, 68, 69] and Tenericutes was lean-associated (*n* = 4) [20, 22, 48, 77] (Table 2). The details on the differential levels of taxon in each eligible study are shown in Supplementary table 1.

**Table 2:** Differentially abundant phyla in obesity/metabolic diseases

No. of studies	3 or more papers with obese/metabolic diseases	2 papers with obese/metabolic diseases	1 paper with obese/metabolic diseases	0 paper with obese/metabolic diseases
3 or more papers with lean/metabolically healthy	Bacteroidetes (8, 12)*	–	–	Tenericutes (4)
	Firmicutes (7, 15)			Actinobacteria (7)
2 papers with lean/metabolically healthy	Proteobacteria (13)	–	Verrucomicrobia	–
1 paper with lean/metabolically healthy			–	Candidatus Saccharibacteria
				Elusimicrobia
				Ignavibacteriae
				Rikenellaceae
				Lentisphaerae
				Prevotellaceae
0 paper with lean/metabolically healthy	Fusobacteria (5)		Acidobacteria	–
			Cyanobacteria	

*n (lean/metabolically healthy, obese/metabolic diseases)

At lower taxonomic levels, studies consistently reported the class Bacilli, Gammaproteobacteria and family Coriobacteriaceae to be obesity-associated. Controversial results were reported for class Clostridia, family Lachnospiraceae, Rikenellaceae, and Ruminococcaceae **(**Supplementary table 2). At the genus level, *Alistipes*, *Akkermansia*, *Bifidobacterium*, *Desulfovibrio*, and genera in the *Clostridium* cluster IV (*Faecalibacterium*, *Eubacterium*, *Oscillospira*, *Odoribacter*) were the most reported lean-associated genera, while *Prevotella*, *Lactobacillus*, *Blautia*, *Escherichia*, *Succinivibrio*, and *Fusobacterium* were the most reported obesity-associated genera. Significant change in genera *Ruminococcus*, *Coprococcus*, *Dialister*, *Bacteroides*, *Clostridium* and *Roseburia* were reported but results were controversial (Table 3).

**Table 3:** Differentially abundant genera in obesity/metabolic diseases

No. of studies	3 or more papers with obesity-associated	2 papers with obesity-associated	1 paper with obesity-associated	0 paper with obesity-associated
3 or more papers with lean-associated	*Faecalibacterium* (13,3) [18–20, 22, 26, 44, 46, 58, 59, 66, 69, 71, 72]	*Bifidobacterium* (6) [20–22, 57, 58, 68]	*Alistipes* (10) [20, 26, 44, 53, 58–60, 68, 76, 77]	*Odoribacter* (6) [29, 44, 59, 60, 77, 78]
	*Prevotella* (5,6) [26, 38, 67, 72, 73, 75]	*Roseburia* (4) [53, 63, 66, 68, 69, 79]	*Akkermansia* (9) [23, 28, 36, 44, 45, 47, 49, 65, 70]	*Oscillospira* (6) [20, 36, 68, 70, 75, 77]
	*Bacteroides* (6,4) [18, 24, 26, 41, 43, 44, 46, 48, 69, 72]	*Clostridium* (4) [20, 38, 46, 49, 53, 72]	*Turicibacter* (3)	*Oscillibacter* (4)
	*Ruminococcus* (4, 5) [20, 23, 39, 44, 49, 62, 63, 68, 69]			*Eubacterium* (3) [20, 44, 68]
	*Dialister* (4,4) [19, 20, 36, 50, 55, 70, 72, 79]			*Desulfovibrio* (3) [18, 20, 44]
	*Lactobacillus* (3,6) [19, 21, 38, 46, 57, 60]			*Anaerotruncus* (3)
	*Coprococcus* (3, 5) [18, 23, 44, 48, 63, 68, 69, 71]			
	*Blautia* (3,6) [38, 39, 44, 48, 73, 74]			
2 papers with lean-associated	*Streptococcus* (4)	*Bilophila*	*Holdemania*	*Oxalobacter*
	*Lachnospira* (3)			*Methanobrevibacter*
	*Fusobacterium* (4) [18, 20, 22, 44]			*Acholeplasma*
				*gemmiger*
1 paper with lean-associated		*Sutterella*	*Veillonella*	
	*Phascolarctobacterium* (3)	*Megasphaera*	*Staphylococcus*	*Haemophilus*
	*Dorea* (4)	*Megamonas*	*Rothia*	*Anaerostipes*
	*Collinsella* (3)	*Adlercreutzia*	*Pseudomonas*	*Parabacteroides*
	*Acidaminococcus* (3)		*Parasutterella*	
			*Lactococcus*	
			*Klebsiella*	
			*Haemophilus*	
0 paper with lean-associated	*Succinivibrio* (3) [38, 69, 78]	*SMB53*	*Alloprevotella*	
	*Escherichia* (3) [57, 60, 68]	*Porphyromonas*	*Lachnospiraceae incertae sedis*	
		*Peptoniphilus*	*Burkholderiales*	
		*Mitsuokella*		
		*Escherichia-Shiguela*		
		*Catenibacterium*		
		*Bacillus*		
		*Aggregatibacter*		

*n (lean-associated, obesity-associated)

For most studies used 16s rRNA sequencing, which lacks species resolution, *Faecalibacterium prausnitzii*, and *Akkermansia muciniphila* were combined with respective genera as they were the primary species that constitute respective genera

### Secondary outcomes

Forty (67%) studies provided alpha diversity of the gut microbiota. Among them, 18 reported significant reduction in diversity while four reported significant increase of alpha diversity in obesity and metabolic disorders compared with controls. The remaining studies (*n* = 18) found no significant difference in alpha diversity between both groups. In addition, 11 studies demonstrated significant difference in β-diversity [20, 23, 27, 28, 32, 40, 47, 55, 58, 66, 69], while 10 studies showed no significant difference in β-diversity between patients with obesity and metabolic disorders and controls [24, 26, 38, 49, 50, 57, 65, 70, 74, 79]. Twenty-two (37%) studies reported Firmicutes/Bacteroidetes (F/B) ratio [51–54, 56–68, 71–75]. Among them, eight studies reported significant increase [34–36, 39, 48, 52, 59, 75] and three studies reported a significant decreased in F/B ratio [33, 41, 44]. Eleven studies reported no significant change in F/B ratio in patients with obesity and metabolic disorders compared with controls (Supplementary Table 3) [37, 42, 46, 53, 54, 60–63, 67, 68].

### Difference of microbiota between adult and childhood obesity

The trend for most microbial changes in adult and childhood obesity were consistent. Studies reported Actinobacteria as lean-associated, while Proteobacteria and Firmicutes as obesity-associated in both adults and childhood obesity. However, discrepancies were observed for several genera. Three studies in adults consistently reported that *Fusobacterium* was obesity-associated, but controversial results were found in children [18, 20, 22, 32, 61, 77]. Moreover, more studies reported that *Dorea* [39, 46, 49, 77] and *Ruminococcus* [39, 44, 49, 69] were obesity-associated in adults, while more studies reported them to be lean-associated in children [19, 68]. Three studies consistently reported that *Turicibacter* was lean-associated in adults [44, 66, 69], but one study reported it to be obesity-associated in children [20]. Notably, three studies in adults reported that the genus *Bifidobacterium* was lean-associated [22, 57, 58], while controversial results were found in children (3 lean-associated and 2 obesity-associated) [19–21, 38, 68]. These findings suggested that microbiota in childhood obesity and metabolic disorders were more heterogeneous compared with adults.

### Difference of microbiota between the East and the West

Large discrepancies in gut microbiome in obesity and metabolic disorders were observed in studies from the East and the West. Four studies exclusively consisting of populations in the West reported that the Family Coriobacteriaceae was obesity-associated [27, 38, 53, 71] whereas none in the East reported significant change of this bacterial family between obese subjects and controls. Four studies in the East reported that the family Ruminococcaceae was lean-associated [22, 60, 61, 63], but conflicting results were found in studies from the West (2 lean-associated and 2 obesity-associated) [27, 36, 43, 68]. At the genus level, four studies reported that *Prevotella* was lean-associated in the East (3 lean-associated and 1 obesity-associated) [19, 20, 26, 61], while other studies from the West have reported it to be obesity-associated (2 lean-associated and 5 obesity-associated) [38, 55, 67, 68, 72, 73, 75]. Three studies reported that *Ruminococcus* was lean-associated in the East [20, 63, 67], but most studies reported it to be obesity-associated in the West (1 lean-associated and 5 obesity-associated) [23, 39, 44, 49, 62, 69]. Similar findings were observed for *Roseburia* (3 lean-associated in the east [30, 63, 66], 1 lean-associated and 2 obesity-associated in the west [53, 68, 69]). Notably, the common genus *Lactobacillus* was repeatedly reported to be obesity-associated in the West (1 lean-associated and 4 obesity-associated) [19, 38, 44, 46, 57]. Controversial results for *Lactobacillus* were also reported in the East (2 lean-associated and 2 obesity-associated) [21, 59, 60, 63].

### Quality of the evidence

The Newcastle Ottawa Scale showed that all 60 studies provided an adequate explanation in the definition and selection method for patients with obesity and metabolic disorders (Table 4). Fifty-five (91.7%) of 60 studies did the same process for controls. Twenty (33.3%) and 27 (45%) studies demonstrated comparable data of sex and age in patients with obesity / metabolic disorders and controls.

**Table 4:** Quality of each included study by the Newcastle Ottawa Scale

First author, year	Selection				Comparability		Exposure		
	Is the case definition adequate?	Representativeness of the cases	Selection of controls	Definition of controls	Comparability of baseline characteristic 1 (sex)	Comparability of baseline characteristic 2 (Age)	Ascertainment of exposure	Same method of ascertainment for cases and controls	Nonresponse rate
Andoh, 2016 [18]	*	*	*	*	NA	NA	NA	NA	*
Bai, 2019 [19]	*	*	NA	NA	NA	NA	NA	NA	*
Chen, 2020 [20]	*	*	*	*	NA	NA	NA	NA	*
Da Silva, 2020 [21]	*	*	*	*	*	*	NA	NA	*
Gao, 2018 [22]	*	*	*	*	NA	NA	NA	NA	*
Gao, 2018 [23]	*	*	*	*	*	*	NA	NA	*
Haro, 2016 [24]	*	*	*	*	*	NA	NA	NA	*
Houttu, 2018 [25]	*	*	*	*	NA	*	NA	NA	*
Hu, 2015 [26]	*	*	*	*	*	*	NA	NA	*
Kaplan, 2019 [27]	*	*	NA	NA	NA	NA	NA	NA	*
Liu, 2017 [28]	*	*	*	*	NA	NA	NA	NA	*
Lopez-Contreras, 2018 [29]	*	*	*	*	*	*	*	NA	*
Lv, 2019 [30]	*	*	NA	NA	NA	NA	NA	NA	*
Mendez-Salazar, 2018 [31]	*	*	*	*	NA	NA	*	NA	*
Nardelli, 2020 [32]	*	*	*	*	NA	NA	NA	NA	*
Blasco, 2017 [33]	*	*	*	*	NA	*	NA	NA	*
Davis, 2017 [34]	*	*	*	NA	NA	NA	*	NA	*
Dominianni, 2015 [35]	*	*	*	*	*	*	NA	NA	*
Escobar, 2015 [36]	*	*	*	*	NA	*	NA	NA	*
Kasai, 2015 [37]	*	*	*	*	*	*	NA	NA	*
Nirmalkar, 2018 [38]	*	*	*	*	NA	*	NA	NA	*
Ottosson, 2018 [39]	*	*	*	*	NA	NA	NA	NA	*
Peters, 2018 [40]	*	*	*	*	*	*	NA	NA	*
Ppatil, 2012 [41]	*	*	*	*	NA	NA	NA	NA	*
Rahat- Rozenbloom, 2014 [42]	*	*	*	*	*	*	NA	NA	*
Riva, 2017 [43]	*	*	*	*	NA	NA	NA	NA	*
Vieira-Silva, 2020 [44]	*	*	*	NA	NA	NA	*	*	*
Ville, 2020 [45]	*	*	*	*	NA	NA	NA	NA	*
Yasir, 2015 [46]	*	*	*	*	NA	NA	NA	NA	*
Yun, 2017 [47]	*	*	*	*	NA	*	NA	NA	*
Zacarias, 2018 [48]	*	*	*	*	NA	*	*	NA	*
Allin, 2018 [49]	*	*	*	*	*	*	NA	NA	*
Barengolts, 2018 [50]	*	*	*	*	NA	*	*	NA	*
Leite, 2017 [51]	*	*	*	*	NA	NA	NA	NA	*
Qin, 2012 [52]	*	*	*	*	NA	NA	NA	NA	*
Karlsson, 2013 [53]	*	*	*	*	NA	NA	NA	NA	*
Larsen, 2010 [54]	*	*	*	*	NA	NA	NA	NA	*
Ahmad, 2019 [55]	*	*	*	*	*	NA	NA	NA	*
Koo, 2019 [56]	*	*	*	*	*	*	NA	NA	*
Sroka-oleksiak, 2020 [57]	*	*	*	*	NA	*	NA	NA	*
Thingholm, 2019 [58]	*	*	*	*	NA	NA	NA	NA	*
Zhao, 2019 [59]	*	*	*	*	NA	NA	NA	NA	*
Jiang, 2018 [60]	*	*	*	*	*	*	NA	NA	*
Shen, 2017 [61]	*	*	*	*	*	*	NA	NA	*
Sobhonslidsuk, 2018 [62]	*	*	*	*	*	*	NA	NA	*
Wang, 2016 [63]	*	*	*	NA	NA	NA	NA	NA	*
Li, 2018 [64]	*	*	*	*	*	*	NA	NA	*
Nistal, 2019 [65]	*	*	*	*	*	*	NA	NA	*
Yun, 2019 [66]	*	*	*	*	*	*	NA	NA	*
Michail, 2015 [67]	*	*	*	*	NA	NA	NA	NA	*
Zhu, 2013 [68]	*	*	*	*	NA	NA	NA	NA	*
Chavez-Carbajal, 2019 [69]	*	*	*	*	NA	*	NA	NA	*
De La Cuesta-Zuluaga, 2018 [70]	*	*	*		NA	NA	*	NA	*
Gallardo-Becerra, 2020 [71]	*	*	*	*	*	*	NA	NA	*
Gozd-Barszczewska, 2017 [72]	*	NA	NA	NA	NA	NA	*	NA	*
Kashtanova, 2018 [73]	*	*	NA	NA	NA	NA	NA	NA	*
Lippert, 2017 [74]	*	*	*	*	NA	NA	NA	NA	*
Feinn, 2020 [75]	*	*	*	*	*	*	NA	NA	*
Li, 2021 [76]	*	*	*	*	NA	NA	NA	NA	*
Yuan, 2021 [77]	*	*	*	*	NA	NA	NA	NA	*

*NA* not appliable

## Discussion

To our knowledge, this is the most comprehensive systematic review in microbiota and obesity and metabolic disorders, as we extracted the data of each available bacterial group using the lowest taxonomic level based on NGS of each included study. We believe that the findings reflect the best available current evidence demonstrating the relationship between individual bacterial taxa and obesity or metabolic disorders.

Proteobacteria was the most consistently reported obesity-associated phylum. Several members of Proteobacteria, such as *Proteus mirabilis* and *E. coli*, were potential drivers of inflammation in the gastrointestinal tract [7, 80, 81]. Low-grade inflammation is a risk factor for developing metabolic diseases including atherosclerosis, insulin resistance, and diabetes mellitus [82]. Besides stool microbiota, obese subjects with T2DM also showed a high bacterial load with an increase in Enterobacteriaceae in plasma, liver, and omental adipose tissue microbiota [83].

*Lactobacillus* was reported to be an obesity-associated taxon and abundance was higher in the stool of patients with obesity and metabolic diseases. This food-derived probiotic genus showed relative low prevalence and abundance in the commensal gut microbiota [52]. Previous clinical trials of *Lactobacillus*, alone or in combination with *Bifidobacterium,* showed variable efficacy in weight loss in patients with obesity [9]. These inconsistent results indicated that the underlying mechanisms of *Lactobacillus* (at least some of its species) in the treatment of metabolic disorders warrant further investigation. Other commensal bacteria such as *Bifidobacterium* spp., *Alistipes* spp., and *Akkermansia* that constitute a large proportion of the gut microbiota were frequently observed to be higher in healthy individuals than obese, metabolically affected subjects. These species might therefore exert a more durable beneficial effect for the consideration in managing obesity compared with *Lactobacillus*.

*Akkermansia muciniphila* (Actinobacteria phylum), a species identified by NGS, was one of the most commonly reported lean-associated bacteria in obesity and metabolic diseases. *A. muciniphila* was reported to help modulate the gut lining which could promote gut barrier function and prevent inflammation caused by the “leaky” gut [84]. A clinical trial demonstrated that supplementation with *A. muciniphila* could reduce body weight and decrease the level of blood markers for liver dysfunction and inflammation in obese insulin-resistant volunteers [10]. Another proof-of-concept study showed that supplementation with five strains including *A. muciniphila* was safe and associated with improved postprandial glucose control [85]. These findings highlight the potential of specific live biotherapeutics in weight control in subjects with obesity and metabolic diseases.

Other genera that were consistently reported to be more abundant in lean healthy individuals than obese subjects were *Alistipes* (Bacteroidetes phylum) and *Faecalibacterium* (Firmicutes phylum). *Alistipes* could produce small amounts of short-chain fatty acids (SCFA, acetic, isobutyric, isovaleric, and propionic acid) [86] while *Faecalibacterium* is one of the major butyrate producers in the human gut [87, 88]. SCFA have anti-inflammatory properties [89] and may promote weight loss through the release of glucagon-like peptide 1 that promotes satiety and the activation of brown adipose tissue via the gut–brain neural circuit [90, 91]. Butyrate could activate the GPR43-AKT-GSK3 signaling pathway to increase glucose metabolism by liver cells and improve glucose control in diabetes mice [92]. They could also inhibit the expression of PPARγ, increase fat oxidation in skeletal muscle mitochondria, and reduce lipogenesis in high-fat diet (HFD) mouse model [93].

We have identified several genera, including *Bifidobacterium*, *Roseburia*, *Prevotella*, and *Ruminococcus,* that were consistently reported to be lean-associated exclusively in subjects from the East. *Bifidobacterium* spp. are widely used probiotics proven to be safe and well-tolerated and exhibited a significant effect in lowering serum total cholesterol both in mice and in humans [94]. *Roseburia* is another major butyrate-producing genus of the human gut [95]. *R. intestinalis* could maintain the gut barrier function through upregulation of the tight junction protein [96]. Supplementation of *R. intestinalis* and *R. hominis* could ameliorate alcoholic fatty liver disease in mice [97]. *Ruminococcus bromii* is a keystone species for the degradation of resistant starch in the human colon [98]. *Prevotella copri* (Bacteroidetes phylum) was found to improve aberrant glucose tolerance syndromes and enhance hepatic glycogen storage in animals via the production of succinate [99]. However, a recent study also showed that the prevalence of *P. copri* exacerbated glucose tolerance and enhanced insulin resistance which occur before the development of ischemic cardiovascular disease and type 2 diabetes [100].

Only limited human studies in the current review reported an increased ratio of F/B in obesity. An increased ratio of F/B was shown in studies of the high-fat diet mouse model [6]. No taxon distinction was found to be specific for any type of metabolic disease. This was in line with a recent study that showed obesity, but not type 2 diabetes, was associated with notable alterations in microbiome composition [58].

The strength of this study is that we applied a robust method of grouping various types of disease-microbiome associations into “lean, metabolically healthy state” or “obese, metabolically diseased state.” Despite various metabolic disorders may affect the gut microbiota in different manners, the inter-study variation often supersedes the intra-study variation between disease and control groups [101]. Overall, the most striking observation is the lack of consistency in results between studies. This probably relates to the limitations of the studies included in this review. Also, it relies on the striking stability and individuality of adult microbiota, changing over time. Heterogeneity between studies is often a problem in systematic reviews. Several different methods were used to assess the microbiota, which makes it difficult to compare results between studies and likely contributes to the differences in results. While the standardization of study protocol (sample storage, DNA extraction, sequencing, analysis methods, and stringent subject recruitment criteria) could potentially result in comparable data between studies, this remains a big challenge across different regions. Moreover, we excluded studies that used species- or group-specific primers for microbiota assessment because such methods could only capture certain bacterial groups. This limits the total number of studies included. For robust microbiota results that are comparable among studies, there need to be efforts for standardization of sample storage, DNA extraction, sequencing, and analysis methods among groups undertaking gut microbiota studies. Finally, longitudinal studies would allow for a more robust association of changes in the microbiota to changes in obesity and metabolic disorders.

## Conclusions

This systematic review identified consistent evidence for several lean-associated genera that may have therapeutic potential for obesity and metabolic diseases. Besides *A. muciniphila*, species from genera *Faecalibacterium*, *Alistipes*, and *Roseburia* might also harbor therapeutic potentials against obesity and metabolic diseases. These results provided a guide for the future development of certain bacteria into live biotherapeutics that may be helpful for the management of obesity and metabolic disorders. Further in-vitro and in-vivo research are needed to elucidate their role in the management of obesity and metabolic diseases.

### Supplementary Information

**Additional file 1.** Supplementary Table 1. Differentially abundant taxa at each taxonomic level in patients with obesity and metabolic diseases reported in individual studies. Supplementary Table 2. Differentially abundant taxa at class, order, and family level in obesity / metabolic diseases. Supplementary Table 3. Microbiota diversity and F/B Ratio in Obesity / metabolic diseases.

## Data Availability

Data sharing not applicable to this article as no datasets were generated or analyzed during the current study.

## Appendix. Searching strategy

1 obese.mp. [mp=ti, ab, hw, tn, ot, dm, mf, dv, kw, fx, dq, nm, kf, ox, px, rx, an, ui, sy]

2 obesity.mp. [mp=ti, ab, hw, tn, ot, dm, mf, dv, kw, fx, dq, nm, kf, ox, px, rx, an, ui, sy]

3 overweight.mp. [mp=ti, ab, hw, tn, ot, dm, mf, dv, kw, fx, dq, nm, kf, ox, px, rx, an, ui, sy]

4 microbiota.mp. [mp=ti, ab, hw, tn, ot, dm, mf, dv, kw, fx, dq, nm, kf, ox, px, rx, an, ui, sy]

5 microbiome.mp. [mp=ti, ab, hw, tn, ot, dm, mf, dv, kw, fx, dq, nm, kf, ox, px, rx, an, ui, sy]

6 fecal.mp. [mp=ti, ab, hw, tn, ot, dm, mf, dv, kw, fx, dq, nm, kf, ox, px, rx, an, ui, sy]

7 faecal.mp. [mp=ti, ab, hw, tn, ot, dm, mf, dv, kw, fx, dq, nm, kf, ox, px, rx, an, ui, sy]

8 gut.mp. [mp=ti, ab, hw, tn, ot, dm, mf, dv, kw, fx, dq, nm, kf, ox, px, rx, an, ui, sy]

9 intestinal.mp. [mp=ti, ab, hw, tn, ot, dm, mf, dv, kw, fx, dq, nm, kf, ox, px, rx, an, ui, sy]

10 1 or 2 or 3

11 4 or 5

12 6 or 7 or 8 or 9

13 10 and 11 and 12

14 metagenomics.mp. [mp=ti, ab, hw, tn, ot, dm, mf, dv, kw, fx, dq, nm, kf, ox, px, rx, an, ui, sy]

15 metagenomic.mp. [mp=ti, ab, hw, tn, ot, dm, mf, dv, kw, fx, dq, nm, kf, ox, px, rx, an, ui, sy]

16 16s.mp. [mp=ti, ab, hw, tn, ot, dm, mf, dv, kw, fx, dq, nm, kf, ox, px, rx, an, ui, sy]

17 14 or 15 or 16

18 13 and 17

19 remove duplicates from 18

20 metabolic disease.mp. [mp=ti, ab, hw, tn, ot, dm, mf, dv, kw, fx, dq, nm, kf, ox, px, rx, an, ui, sy]

21 10 or 20

22 11 and 12 and 21

23 17 and 22

24 remove duplicates from 23

25 limit 24 to full text

## Abbreviations

OB: Obesity
T2DM: Type 2 diabetes
NAFLD: Non-alcoholic fatty liver disease
NGS: Next-generation sequencing
BMI: Body mass index
F/B: Firmicutes/Bacteroidetes
NASH: Non-alcoholic steatohepatitis
OMS: Obese with metabolic syndrome
SCFA: Short-chain fatty acids
HFD: High-fat diet
