# Supplementary material for: Gut microbiota in patients with obesity and metabolic disorders — a systematic review
Source: Genes Nutr. 2022 Jan 29;17:2. doi: 10.1186/s12263-021-00703-6 (PMC8903526; doi:10.1186/s12263-021-00703-6)
Supplement: Supplementary file 1 — Additional file 1. Supplementary Table 1. Differentially abundant taxa at each taxonomic level in patients with obesity and metabolic diseases reported in individual studies. Supplementary Table 2. Differentially abundant taxa at class, order, and family level in obesity / metabolic diseases. Supplementary Table 3. Microbiota diversity and F/B Ratio in Obesity / metabolic diseases. [file 12263_2021_703_MOESM1_ESM.docx]

Additional files

Supplementary Table 1. Differentially abundant taxa at each taxonomic level in patients with obesity and metabolic diseases reported in individual studies

| First author, year | Phylum | Class | Order | Family | Genus |
| --- | --- | --- | --- | --- | --- |
| Andoh, 2016^16^ | Firmicutes↑;  Fusobacteria↑ | NA | Bacteroidales↓; Clostridiales↑ | NA | Bacteroides ↓; Desulfovibrio↓; Faecalibacterium↓; Lachnoanaerobaculum↓; Olsenella↓; Alistipes↑; Anaerococcus↑; Corpococcus↑; Fusobacterium↑; Parvimonas↑ |
| Bai, 2019^17^ | Proteobacteria↑ | NA | NA | NA | Dorea↓; Faecalibacterium↓; Prevotella↓; Rikenellaceae_Family_Unclassified↓; Sutterella↓; Acinetobacter↑; Bacillus↑; Bifidobacterium↑; Dialister↑; Lactobacillus↑ |
| Chen, 2020^18^ | Bacteroidetes↓; Actinobacteria↓; Tenericutes↓; Fusobacteria↑; Proteobacteria↑ | Deltaproteobacteria↓;  Erysipelotrichia↓; Bacilli↑ | NA | Barnesiellaceae↓; Paraprevotellaceae↓; Rikenellaceae↓; S24-7↓ | Adlercreutzia↓; Alistipes↓; Anaerotruncus↓; Bifidobacterium↓;Bilophila↓;Christensenella↓; Clostridium↓;Collinsella↓;Defluviitalea↓;Desulfovibrio↓; Dialister↓;Eubacterium↓;Holdemania↓;Oscillospira↓;Oxalobacter↓;Prevotella↓;Ruminococcus↓;Sporobacter↓;Actinobacillus↑;Aggregatibacter↑;Campylobacter↑;Faecalibacterium↑;Fusobacterium↑;Granulicatella↑;Haemophilus↑;Lachnospira↑;Megamonas↑;Phascolarctobacterium↑;Rothia↑;SMB53↑;Streptococcus↑;Sutterella↑;Turicibacter↑;Veillonella↑ |
| Da Silva, 2020^19^ | Firmicutes↑ | NA | NA | Bifidobacteriaceae↓ | Bifidobacterium↓; Lactobacillus↑ |
| Gao, 2018^20^ | Tenericutes↓; Fusobacteria↑; Proteobacteria↑ | NA | NA | Ruminococcaceae↓ | Bifidobacterium↓; Faecalibacterium↓; Ruminococcaceae_UCG-002↓; Ruminococcaceae_UCG003↓; Ruminococcaceae_UCG-005↓; Ruminococcaceae_UCG-010↓; Ruminococcaceae_UCG-014↓; Bacillus↑; Escherichia/Shigella↑; Fusobacterium↑; Pseudomonas↑ |
| Gao, 2018^21^ | Actinobacteria↓; Candidatus_Saccharibacteria↓; Firmicutes↓; Verrucomicrobia↓; Bacteroidetes↑ | NA | NA | NA | NA |
| Haro, 2016^22^ | NA | NA | NA | NA | Bacteroides↑ |
| Houttu, 2018^23^ | NA | NA | NA | Prevotellaceae↑ | NA |
| Hu, 2015^24^ | NA | NA | NA | NA | Bacteroides↓;Alistipes↓;Faecalibacterium↓; Oscillibacter↓;Prevotella↑ |
| Kaplan, 2019^25^ | Cyanobacteria↑ | NA | Clostridiales↑ | Christensenellaceae↑;Coriobacteriaceae↑; Ruminococcaceae↑;Victivallaceae↑ | Acidaminococcus↑;Megasphaera↑ |
| Liu, 2017^26^ | Firmicutes↑ | NA | NA | NA | NA |
| Lopez-Contreras, 2018^27^ | Firmicutes↓;Proteobacteria↑; Bacteroidetes↑; | NA | NA | Christensenellaceae↓ | Odoribacter↓ |
| Lv, 2019^28^ | NA | NA | NA | Lachnospiraceae↓ | Anaerotruncus↓;Blautia↓;Dialister ↓;Klebsiella↓;Megasphaera↓; Roseburia↓;uncultured_Lachnospiraceae↓;Parasuterella↑ |
| Mendez-Salazar, 2018^29^ | Bacteroidetes↓;Proteobacteria↑ | NA | NA | NA | Bilophila↑ |
| Nardelli, 2020^30^ | Actinobacteria↓;Firmicutes↓;Acidobacteria↑; Fusobacteria↓;Proteobacteria↑;Bacteroidetes↑; | NA | Pseudomonadales↑ | NA | NA |
| Blasco, 2017^31^ | Ignavibacteriae↓ | NA | NA | NA | NA |
| Davis, 2017^32^ | Firmicutes↑;Verrucomicrobia↑ | NA | NA | NA | NA |
| Dominianni, 2015^33^ | Bacteroidetes↓ | NA | NA | NA | NA |
| Escobar, 2015^34^ | NA | NA | Clostridiales↓ | Ruminococcaceae↓ | Dialister↓;Oscillospira↓;Akkermansia↓ |
| Kasai, 2015^35^ | Bacteroidetes↓ | NA | NA | NA | NA |
| Nirmalkar, 2018^36^ | NA | NA | Clostridia_SHA_98↓;Solirubrobacterales↓; Streptophyta↑;Bacteroidales↑ | Conexibacteraceae↓;Lactobacillaceae↓;Coriobacteriaceae↑;Elusimicrobiaceae↑;Gemellaceae↑;Leuconostocaceae↑;Mogibacteriaceae↑;Peptostreptococcaceae↑;Veillonellaceae↑ | Acholeplasma↓;Anaerovibrio↓;Fusobacterium↓; Lachnospira↓;Megamonas↓;Nocardioides↓;Paludibacter↓; Adlercreutzia↑;Bifidobacterium↑;Blautia↑;Bradyrhizobiom↑; Candidatus_Portiera↑;Clostridium↑;Collinsella↑;Coprococcus↑; Dickeya↑;Gemella↑;Lactobacillus↑;Lactococcus↑;Prevotella↑; Propiohicimonas↑;Ruminococcus↑;SMB53↑;Stenotrophomonas↑;Succinivibrio↑ |
| Ottosson, 2018^37^ | NA | NA | NA | NA | Blautia↑;Dorea↑;Ruminococcus↑;SHA-98↓ |
| Peters, 2018^38^ | NA | NA | NA | Christensenellaceae↓; Clostridiaceae↓;Dehalobacteriaceae↓; Actinomycetaceae↑;Enterobacteriaceae↑;Gemellaceae↑;Lactobacillaceae↑;Pasteurellaceae↓;Rikenellaceae↓;SHA-98↓;Streptococcaceae↑;Veillonellaceae↑ | Bacilli↑ |
| Ppatil, 2012^39^ | NA | NA | NA | NA | Bacteroides↑ |
| Rahat- Rozenbloom, 2014 ^40^ | Firmicutes↑ | NA | NA | NA | NA |
| Riva, 2017^41^ | Bacteroidetes↓;Firmicutes↑ | NA | NA | Ruminococcaceae↑; Bacteroidaceae↓ | Bacteroides↓ |
| Vieira-Silva, 2020^42^ | NA | NA | NA | NA | Bacteroides↓;Akkermansia↓;Alistipes↓; Butyrivibrio↓;Capnocytophaga↓;Coprococcus↓;Desulfovibrio↓;Eubacterium↓;Faecalibacterium↓;Haemophilus↓;Holdemania↓;Lactobacillus↓;Methanobrevibacter↓;Methanosphaera↓;Odoribacter↓;Oxalobacter↓;Pseudomonas↓;Rothia↓;Streptococcus↓;Turicibacter↓;Veillonella↓;Victivallis↓;Acidaminococcus↑;Aggregatibacter↑;Anaerostipes↑;Atopobium↑;Blautia↑;Catonella↑;Clostridiales↑;Collinsella↑;Eggerthella↑;Fusobacterium↑;Gardnerella↑;Megasphaera↑;Mitsuokella↑;Oribacterium↑;Peptoniphilus↑;Propionibacterium↑;Pyramidobacter↑;Ruminococcus↑ |
| Ville, 2020^43^ | Firmicutes↑; Actinobacteria↓; Proteobacteria↓ | NA | NA | NA | Akkermansia↓(delete) |
| Yasir, 2015^44^ | Proteobacteria↑;Bacteroidetes↑;Firmicutes↑; | NA | NA | NA | Lactobacillus↑;Escherichia-Shiguela↑;Bacteroides↑; Clostridium↓;Faecalibacterium↓;Dorea↑ |
| Yun, 2017^45^ | NA | NA | Bacteroidales↑ | NA | Akkermansia↓; Clostridiales_unknown_genus↓; Acidaminococcus↑;Adlercreutzia↑;Mitsuokella↑;Paraprevotellaceae↑ |
| Zacarias, 2018^46^ | Tenericutes↓; Firmicutes↑ | NA | NA | Bacteroidaceae↓;Desulfovibrionaceae↓; Actinomycetaceae↑;Lachnospiraceae↑ | Bacteroides↓;Actinomyces↑;Blautia↑; Catenibacterium↑;Coprococcus↑ |
| Allin, 2018^48^ | NA | NA | Clostridiales↓ | NA | Clostridium↓;Dorea↑;Ruminococcus↑; Streptococcus↑;Sutterella↑ |
| Barengolts, 2018^49^ | Firmicutes↑ | NA | NA | NA | Dialister↑;Lachnospira↑ |
| Leite, 2017^50^ | NA | NA | NA | NA | only species |
| Qin, 2012^51^ | NA | NA | NA | NA | only species |
| Karlsson, 2013^52^ | Firmicutes↑ | NA | Clostridiales↓ | Coriobacteriaceae↑; Lachnospiraceae↓ | Roseburia↑;Clostridium↑; Alistipes↓ |
| Larsen, 2010^53^ | Firmicutes↓ | Clostridia↓;Betaproteobacteria↑; Clostridia↓ | NA | NA | NA |
| Ahmad, 2019^54^ | Bacteroidetes↓;Elusimicrobia↓; Proteobacteria↓;Verrucomicrobia↓;Firmicutes↑ | Clostridia↑;Coriobacteriia↑; Negativicutes↑ | NA | NA | Prevotella↓;Allisonella↑;Dialister↑; Eubacterium_coprostanoligenes_group↑ |
| Koo, 2019^55^ | NA | NA | NA | Gemellaceae↓;Rikenellaceae↓; Streptococcaceae↓ | Anaerofilum↓ |
| Sroka-oleksiak, 2020^56^ | Bacteroidetes↑ | Gammaproteobacteria↑ | Enterobacteriales↑ | NA | Bifidobacterium↓;Staphylococcus↑; Escherichia↑;Lactobacillus↑ |
| Thingholm, 2019^57^ | NA | NA | NA | NA | Alistipes↓;Bifidobacterium↓;Faecalibacterium↓;Oscillibacter↓ |
| Zhao, 2019^58^ | Bacteroidetes↓;Firmicutes↑; Proteobacteria↑ | Gammaproteobacteria↑ | Enterobacterales↑; Selenomonadales↑ | Lactobacillaceae↓;Odoribacteraceae↓;Oscillospiraceae↓;Rikenellaceae↓;Enterobacteriaceae↑;Selenomonadaceae↑ | Alistipes↓;Faecalibacterium↓;Helicobacter↓;Lactobacillus↓;Odoribacter↓;Oscillibacter↓;Paraprevotella↓;Ruminiclostridium↓;Klebsiella↑;Kluyvera↑;Negativicutes↑;Phascolarctobacterium↑ |
| Jiang, 2018^59^ | Lentisphaerae↓ | NA | NA | Ruminococcaceae↓ | Clostridium XI↑;Anaerobacter↑; Streptococcus↑;Lactobacillus↑;Oscillibacter↓;Flavonifractor↑;Odoribacter↓;Alistipes↓;Escherichia↑ |
| Shen, 2017^60^ | Proteobacteria↑;Fusobacteria↑; Bacteroidetes↓; | NA | NA | Ruminococcaceae↓ | Prevotella↓ |
| Sobhonslidsuk, 2018^61^ | Actinobacteria↓;Bacteroidetes↑;Firmicutes↓ | NA | NA | NA | Ruminococcus↓; Phascolarctobacterium ↑ |
| Wang, 2016^62^ | Bacteroidetes↑;Firmicutes↓ | Bacteroidia↑; Clostridia↑ | NA | Lachnospiraceae↓;Ruminococcaceae↓;Lactobacillaceae↓;Peptostreptococcaceae↓ | Pseudobutyrivibrio↓;Anaerotruncs↓;Lactobacillus↓;Roseburia↓;Coprococcus↓;Ruminococcus↓;Moryella↓;Anaerosporabacter↓ |
| Li, 2018^63^ | NA | NA | NA | Coprobacillaceae↑;Erysipelotrichaceae↑; EtOH8↑;Lactobacillaceae↑;Peptostreptococcaceae↑;Veillonellaceae↑;Victivallaceae↑ | Odoribacter↓;Proteus ↓; Porphyromonas↑;Succinivibrio↑ |
| Nistal, 2019^64^ | Proteobacteria↑ | Bacilli↑ | NA | NA | Akkermansia↓;Alkaliphilus↓; Blautia↓;Flavobacterium↓;Streptococcus↑ |
| Yun, 2019^65^ | NA | NA | NA | Desulfovibrionaceae↓; Enterobacteriaceae↓ | Acidaminococcus↓;Biophila↓; Citrobacter↓;Erysipelothrix↓;Faecalibacterium↓;Fastidiosipila↓;Parasutterella↓;Roseburia↓;Turicibacter↓ |
| Michail, 2015^66^ | NA | Gammaproteobacteria↑;Epsilonproteobacteria ↑ | NA | NA | Prevotella↑ |
| Zhu, 2013^67^ | Actinobacteria↓;Bacteroidetes↑; Prevotellaceae↑;Rikenellaceae↓;Firmicutes↓;Proteobacteria↑ | NA | NA | Bifidobacteriaceae↓;Lachnospiraceae↓; Ruminococcaceae↓; Alcaligenaceae↑;Enterobacteriaceae↑ | Bifidobacterium↓;Prevotella↑;Alistipes↓;Peptoniphilus↑;Blautia↓;Coprococcus↓;Eubacterium↓; Roseburia↓;Oscillospira↓;Ruminococcus↓;Escherichia↑ |
| Chavez-Carbajal, 2019^69^ | Actinobacteria↓;Bacteroidetes↓ ;Proteobacteria↑;Firmicutes↑; |  |  | Erysipelotrichaceae↓;S24–7↑; Lachnospiraceae↑ | Bacteroides↓;Lactococcus↓;Parabacteroides↓; Staphylococcus↓;Streptococcus↓;Turicibacter↓;Roseburia↑;Succinivibrio↑;Coprococcus↑;Faecalibacterium↑;Lachnospira↑;Megamonas↑;Ruminococcus↑ |
| De La Cuesta-Zuluaga, 2018^70^ | NA | NA | NA | Lachnospiraceae↑ | Dialister↓;Methanobrevibacter↓; Oscillospira↓ |
| Gallardo-Becerra, 2020^71^ | Bacteroidetes↓;Firmicutes↑; Proteobacteria↑ | Bacteroidia↓;Bacilli↑; Clostridia↑;Coriobacteria↑;Erysipelotrochi↑ | Clostridiales↑;Coriobacteriales↑; Erysipelotrichales↑ | Coriobacteraceae↑; Erysipelotrichaceae↑ | Bacteroidales↓;Phascolarctobacterium↓; Bifidobacterium_adolescentis↑;Faecalibacterium↑;Parabacteroides_distasonis↑;Porphyromonas↑;Catenibacterium↑;Collinsella↑;Coprococcus↑ |
| Gozd-Barszczewska, 2017^72^ | NA | NA | NA | NA | Bacteroides↓;Clostridium↓; Faecalibacterium↓;Dialister↑;Prevotella↑ |
| Kashtanova, 2018^73^ | NA | NA | NA | NA | Blautia↑;Prevotella↑;Serratia↑ |
| Lippert, 2017^74^ | Bacteroidetes ↓ | Clostridia ↓ | NA | Bacteroidaceae↓;Rikenellaceae ↓; Veillonellaceae↓;Erysipelotrichaceae↑;Lachnospiraceae↑; | Blautia↑;Holdemania↑ |
|  |  |  |  |  |  |
| Feinn, 2020^68^ | Bacteroidetes ↓ | NA | NA | NA | Prevotella ↓; Gemmiger ↓; Oscillospira ↓ |
| Li, 2021^47^ | NA | Clostridia ↓; Deltaproteobacteria↓ | Clostridiales ↓ | Rikenellaceae ↓ Pasteurellaceae ↓ | Alistipes ↓; Haemophilus ↓; Alloprevotella ↑; Lachnospiraceae incertae sedis ↑; Bilophila ↑; Burkholderiales ↑ |
| Yuan, 2021^75^ | Tenericutes ↓ | Deltaproteobacteria ↑; Mollicutes ↑ | Desulfovibrionales ↑; RF39 ↑; family Christensenellaceae ↑; Odoribacteraceae ↑; Porphyromonadaceae ↑,Ruminococcaceae ↑ | NA | Anaerostipes ↓; Alistipes ↓; Desulfovibrio ↓; Fusobacterium ↓; Gemmiger ↓; Odoribacter ↓; Oscillospira ↓; Parabacteroides ↓; Dorea ↑ |

Supplementary Table 2. Differentially abundant taxa at class, order, and family level in obesity / metabolic diseases

| Variable | 3 or more papers with obese/metabolic diseases | 2 papers with obese/metabolic diseases | | 1 paper with obese/metabolic diseases | 0 paper with obese/metabolic diseases |
| --- | --- | --- | --- | --- | --- |
| 3 or more papers with lean/metabolically healthy | Clostridiales (3, 4)* | |  | Rikenellaceae (5)^20,40,49,57,60,76^ |  |
|  | Lactobacillaceae (6, 7) | |  |  |  |
|  | Lachnospiraceae(4,4)^48,54,64,69,71,72,76,78^ | |  |  |  |
|  | Clostridia (3,3)^49,55,56,64,73,76^ | |  |  |  |
|  | Ruminococcaceae(6,3)^22,27,36,43,61,62,64,69,77^ | |  |  |  |
| 2 papers with lean or metabolically healthy |  | | Christensenellaceae | Bacteroidaceae | Bifidobacteriaceae |
|  |  | |  | Desulfovibrionaceae |  |
|  |  | |  |  |  |
| 1 paper with lean/metabolically healthy | Veillonellaceae (3) | | Bacteroidales | Erysipelotrochi |  |
|  | Erysipelotrichaceae (3) | | Peptostreptococcaceae | Bacteroidia | Solirubrobacterales |
|  | Enterobacteriaceae (3) | | Gemellaceae | Streptococcaceae | Clostridia_SHA_98 |
|  |  | |  | S24–7 | Paraprevotellaceae |
|  |  | |  | Pasteurellaceae | Oscillospiraceae |
|  |  | |  | Deltaproteobacteria | Dehalobacteriaceae |
|  |  | |  | Odoribacteraceae | Conexibacteraceae |
|  |  | |  |  | Clostridiaceae |
|  |  | |  |  | Barnesiellaceae |
|  |  | |  |  | Deltaproteobacteria |
|  |  | |  |  |  |
| 0 paper with lean/metabolically healthy | Gammaproteobacteria(3)^58,60,68^ | | Enterobacteriales | Negativicutes |  |
|  | Coriobacteriia (3) | | Victivallaceae | Epsilonproteobacteria | |
|  | Bacilli (3)^20,66,73^ | | Actinomycetaceae | Betaproteobacteria |  |
|  | Coriobacteriaceae (4)^27,38,54,73^ | |  | Streptophyta |  |
|  |  | |  | Selenomonadales |  |
|  |  | |  | Pseudomonadales |  |
|  |  | |  | Erysipelotrichales |  |
|  |  | |  | Coriobacteriales |  |
|  |  | |  | SHA-98 |  |
|  |  | |  | Selenomonadaceae |  |
|  |  | |  | Prevotellaceae |  |
|  |  | |  | Mogibacteriaceae |  |
|  |  | |  | Leuconostocaceae |  |
|  |  | |  | EtOH8 |  |
|  |  | |  | Elusimicrobiaceae |  |
|  |  | |  | Coprobacillaceae |  |
|  |  | |  | Alcaligenaceae |  |
|  |  | |  | Mollicutes |  |
|  |  | |  | RF39 |  |
|  |  | |  | Porphyromonadaceae |  |

* n (lean/metabolically healthy, obese/metabolic diseases)

Supplementary Table 3. Microbiota diversity and F/B Ratio in Obesity / metabolic diseases

| **First Author, year** | **Disease** | **α-diversity** | **β-diversity** | **Increase of F/B ratio** |
| --- | --- | --- | --- | --- |
| Andoh, 2016^16^ | OB | No difference | Significant difference | NA |
| Bai, 2019^17^ | OB | No difference | No difference | NA |
| Chen, 2020^18^ | OB | NA | NA | NA |
| Da Silva, 2020^19^ | OB | No difference | NA | NA |
| Gao, 2018^20^ | OB | NA | No difference | NA |
| Gao, 2018^21^ | OB | Obesity-associated | Significant difference | NA |
| Haro, 2016^22^ | OB | Lean-associated | Significant difference | NA |
| Houttu, 2018^23^ | OB | NA | NA | NA |
| Hu, 2015^24^ | OB | Lean-associated | No difference | NA |
| Kaplan, 2019^25^ | OB | Obesity-associated | NA | NA |
| Liu, 2017^26^ | OB | NA | NA | NA |
| Lopez-Contreras, 2018^27^ | OB | Lean-associated | Significant difference | NA |
| Lv, 2019^28^ | OB | NA | NA | NA |
| Mendez-Salazar, 2018^29^ | OB | No difference | No difference | NA |
| Nardelli, 2020^30^ | OB | NA | NA | NA |
| Blasco, 2017^31^ | OB | Lean-associated | Significant difference | NA |
| Davis, 2017^32^ | OB | No difference | NA | NA |
| Dominianni, 2015^33^ | OB | Lean-associated | No difference | NA |
| Escobar, 2015^34^ | OB | Lean-associated | NA | NA |
| Kasai, 2015^35^ | OB | No difference | NA | NA |
| Nirmalkar, 2018^36^ | OB | No difference | No difference | NA |
| Ottosson, 2018^37^ | OB | Lean-associated | Significant difference | NA |
| Peters, 2018^38^ | OB | Lean-associated | Lean-associated | NA |
| Ppatil, 2012^39^ | OB | No difference | No difference | NA |
| Rahat- Rozenbloom, 2014 ^40^ | OB | NA | No difference | NA |
| Riva, 2017^41^ | OB | NA | NA | NA |
| Vieira-Silva, 2020^42^ | OB | No difference | No difference | NA |
| Ville, 2020^43^ | OB | No difference | NA | NA |
| Yasir, 2015^44^ | OB | Lean-associated | Significant difference | NA |
| Yun, 2017^45^ | OB | NA | NA | NA |
| Zacarias, 2018^46^ | OB | NA | NA | NA |
| Allin, 2018^48^ | T2DM | Lean-associated | Significant difference | NA |
| Barengolts, 2018^49^ | T2DM | Obesity-associated | NA | NA |
| Leite, 2017^50^ | T2DM | NA | NA | Lean-associated |
| Qin, 2012^51^ | T2DM | Obesity-associated | NA | No difference |
| Karlsson, 2013^52^ | T2DM | NA | NA | No difference |
| Larsen, 2010^53^ | T2DM | No difference | NA | No difference |
| Ahmad, 2019^54^ | T2DM | No difference | No difference | NA |
| Koo, 2019^55^ | T2DM | NA | NA | Lean-associated |
| Sroka-oleksiak, 2020^56^ | T2DM | Lean-associated | NA | Obesity-associated |
| Thingholm, 2019^57^ | T2DM | No difference | NA | Lean-associated |
| Zhao, 2019^58^ | NAFLD | Lean-associated | NA | No difference |
| Jiang, 2018^59^ | NAFLD | No difference | NA | No difference |
| Shen, 2017^60^ | NAFLD | Lean-associated | NA | No difference |
| Sobhonslidsuk, 2018^61^ | NASH | No difference | NA | No difference |
| Wang, 2016^62^ | NAFLD | Lean-associated | NA | No difference |
| Li, 2018^63^ | NAFLD | NA | NA | Obesity-associated |
| Nistal, 2019^64^ | NAFLD | NA | NA | Obesity-associated |
| Yun, 2019^65^ | NAFLD | NA | NA | No difference |
| Michail, 2015^66^ | NAFLD | NA | NA | No difference |
| Zhu, 2013^67^ | NASH | NA | NA | No difference |
| Chavez-Carbajal, 2019^69^ | MS | Lean-associated | Significant difference | NA |
| De La Cuesta-Zuluaga, 2018^70^ | MS | Lean-associated | Significant difference | NA |
| Gallardo-Becerra, 2020^71^ | MS | NA | NA | Obesity-associated |
| Gozd-Barszczewska, 2017^72^ | MS | No difference | NA | Obesity-associated |
| Kashtanova, 2018^73^ | MS | No difference | NA | Obesity-associated |
| Lippert, 2017^74^ | MS | Lean-associated | NA | Obesity-associated |
| Feinn, 2020^68^ | NAFLD | No difference | NA | Obesity-associated |
| Li, 2021^47^ | OB | NA | NA | NA |
| Yuan, 2021^75^ | MS | Lean-associated | NA | NA |

OB, obesity; NAFLD, non-alcoholic fatty liver disease; F/B ratio, Firmicutes/Bacteroidetes Ratio; T2DM, type 2 diabetes mellitus; NA, not appliable.
